# Predictive Biomarkers in Thyroid Cancer

**DOI:** 10.3389/fonc.2022.901004

**Published:** 2022-05-06

**Authors:** Elisabetta Macerola, Anello Marcello Poma, Paola Vignali, Agnese Proietti, Clara Ugolini, Liborio Torregrossa, Alessio Basolo, Rossella Elisei, Ferruccio Santini, Fulvio Basolo

**Affiliations:** ^1^ Department of Surgical, Medical, Molecular Pathology and Critical Area, University of Pisa, Pisa, Italy; ^2^ Department of Clinical and Experimental Medicine, University of Pisa, Pisa, Italy

**Keywords:** thyroid cancer, molecular marker, predictive marker, targeted therapy, molecular pathology

## Abstract

In molecular pathology, predictive biomarkers identify which patients are likely to respond to targeted drugs. These therapeutic agents block specific molecules directly involved in cancer growth, dedifferentiation and progression. Until few years ago, the only targeted drugs available for advanced thyroid cancer included multi-tyrosine kinase inhibitors, mainly targeting the MAPK pathway and the angiogenic signaling. The administration of these drugs does not necessarily require a molecular characterization of tumors to assess the presence of predictive alterations. However, the availability of new selective targeted drugs for thyroid cancer patients is changing the diagnostic strategies for the molecular characterization of these tumors. The search for targetable alterations can be performed directly on tumor tissue by using a variety of methodologies, depending also on the number and type of alterations to test (i.e. single nucleotide variation or gene rearrangement). Herein, a comprehensive review of the currently available targeted treatments for thyroid cancer, related predictive markers and testing methodologies is provided.

## Introduction

**Figure 1:**
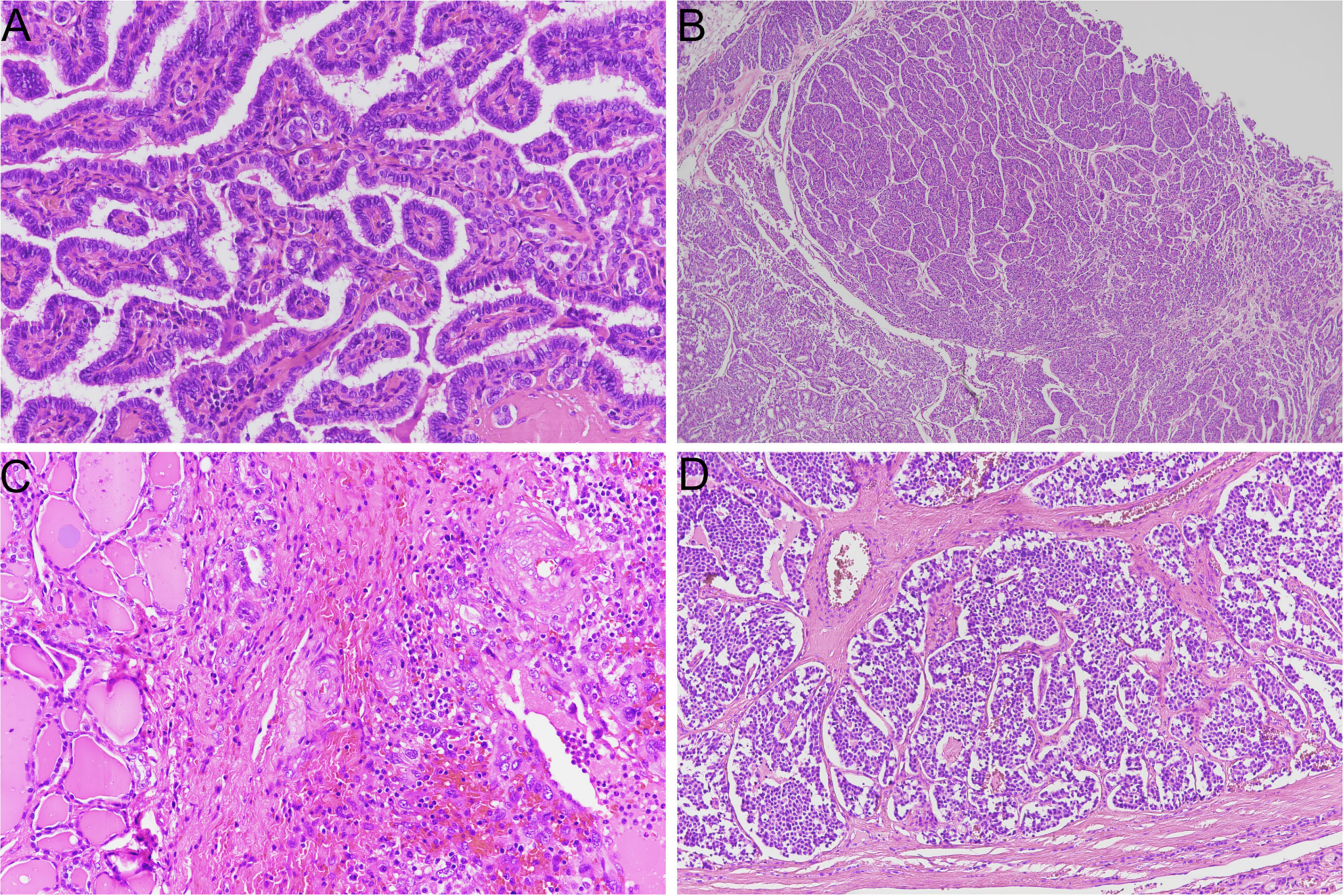
Representative histological slides of thyroid cancer subtypes (hematoxylin/eosin stain). **(A)** papillary thyroid carcinoma (original magnification 20X); **(B)** poorly differentiated thyroid carcinoma with insular growth pattern (original magnification 4X); **(C)** anaplastic thyroid carcinoma (original magnification 20X); **(D)** medullary thyroid carcinoma (original magnification 10X).

Thyroid cancer includes several histo-pathological entities, which are characterized by different histological, molecular, and clinical features. Carcinomas that originate from parafollicular cells, i.e. medullary thyroid carcinoma (MTC), should be distinguished from those derived from follicular cells. Medullary and non-medullary thyroid cancers are characterized by profound differences in terms of morphology, molecular landscape, clinical staging, and treatment (**Figure 1**).

A major distinction of thyroid carcinoma derived from follicular cells can be made depending on the degree of differentiation; these tumors can be classified as well-differentiated (WDTC), poorly differentiated (PDTC) and anaplastic thyroid carcinoma (ATC) (1). WDTCs include papillary thyroid carcinoma (PTC), follicular thyroid carcinoma (FTC) and Hürthle cell carcinoma (HCC). Each histotype presents peculiar morphological and molecular characteristics. Moreover, treatment strategies and prognosis could differ considerably among tumor types. PTC is the most common endocrine malignancy, and its standard treatment (thyroidectomy followed or not by radioiodine ablation) represents a definitive cure for most patients; 10-year overall survival is higher than 90% (2, 3). On the other hand, ATC is rare and frequently shows locally advanced disease and metastatic spread; survival of patients with ATC is dramatically short, with an overall survival after diagnosis of six months (4).

Until few years ago, targeted treatment options for advanced and metastatic disease included only nonselective tyrosine kinase inhibitors (TKI) (5). These agents block multiple tyrosine kinases, mostly participating to the mitogen-activated protein kinase (MAPK) pathway, frequently upregulated in thyroid cancer. Moreover, nonselective TKI have anti-angiogenic effects, targeting molecules as the vascular endothelial growth factor (VEGF) receptors. The administration of these drugs is independent of the presence of specific somatic molecular alterations.

The landscape of targeted treatments for thyroid cancer has been recently expanded. Agents that selectively block altered *RET* and *NTRK* receptors have been approved by the US Food and Drug Administration (FDA) and the European Medicines Agency (EMA) (6). The search for predictive biomarkers has then become mandatory in thyroid cancer patients eligible for treatment.

The aim of this review is to provide a comprehensive view of predictive biomarkers that currently guide thyroid cancer patient management, taking into account the histological, molecular and clinical differences among thyroid tumor types.

## Thyroid Cancer: Histotypes to Be Screened for Predictive Biomarkers

In this section, a summary of thyroid cancer histological patterns, molecular aspects, and therapeutic protocols is provided to give an idea of which tumors deserve molecular screening for predictive purposes.

### Well-Differentiated Thyroid Carcinoma (WDTC)

Among follicular-derived tumors, WDTC are common, with PTC being the most frequent malignancy affecting the thyroid gland (1). A pre-operative diagnosis of WDTC can be made by considering ultrasound and cytological findings. Then, the standard treatment protocol for this tumor includes a surgical removal of the lobe or the entire gland, with or without central and lateral neck dissection. After a post-operative assessment of disease status, radioactive iodine (RAI) therapy for minimizing the risk of disease recurrence can be either considered or not (2). The great majority of patients show an excellent response to standard treatments. Nevertheless, some WDTC patients do not respond or develop resistance to RAI therapy (3, 4). These patients frequently show aggressive tumors in terms of histo-morphology, loco-regional invasiveness, and clinical stage. For example, there are histological variants known to confer a more unfavorable outcome, such as the tall cell and the hobnail variants of PTC, and widely invasive FTC/HCC (1, 7). Although WDTC are generally characterized by a slow progression, RAI resistant advanced tumors can suddenly become locally aggressive and prone to metastatic spread and dedifferentiation (8).

The molecular landscape of WDTC deserves separate definitions for PTC, FTC, and HCC. In PTC, the most frequently encountered molecular alteration is by far the *BRAF* p.V600E mutation (*BRAF* ^V600E^), detected in 45-50% of cases (9). However, *BRAF* ^V600E^ prevalence largely varies across PTC variants. In tall cell and hobnail variants, *BRAF* ^V600E^ can be present in more than 70-80% of cases; in follicular variant PTC, in less than 10% (10, 11). Based on genomic and transcriptomic data, The Cancer Genome Atlas (TCGA) study on PTC highlighted that they can be distinguished into *BRAF* ^V600E^-like and *RAS*-like tumors. *BRAF* ^V600E^-like tumors harbor also *RET* fusions, while *RAS*-like tumors show *RAS* mutations, the *BRAF* ^K601E^, and *PPARG* and *THADA* fusions (12).

By extending this definition to other WDTCs, FTCs can be considered as *RAS*-like tumors. In fact, FTCs show a high prevalence of *RAS* mutations (13). HCCs have a different set of genetic alterations, including also mitochondrial DNA mutations and aneuploidies (14, 15). In WDTCs, secondary mutations (i.e. *TP53* and *TERT* promoter mutations) are generally detected with a low frequency, which become higher in aggressive variants and in advanced stage, RAI resistant and metastatic tumors (9).

Biomarkers able to predict RAI responsiveness of WDTC have been proposed. In particular, *TERT* promoter mutations, especially when detected in co-occurrence with *BRAF* or *RAS* mutations, have been associated to RAI resistance (16–18). However, no molecular testing to guide RAI therapy administration is currently recommended by clinical guidelines.

On the contrary, in the advanced setting, patients are more likely to become potentially eligible for treatment with targeted drugs, therefore they should be screened for predictive molecular alterations. Although there is controversy on the optimal timing for performing molecular testing, a molecular screening of all WDTCs, independently of the therapeutic implications, is not currently recommended (4).

In practical terms, based on the molecular hallmarks, FTC and HCC are subtypes with a very limited set of targetable alterations (19). In fact, in these tumors, the *BRAF* ^V600E^ is virtually absent, and actionable rearrangements are extremely rare, independently of the tumor stage and aggressiveness. On the other hand, PTCs harbor more frequently targetable alterations, but these are restricted to *BRAF* and *RET* fusions and mostly limited to classic and tall cell subtypes (20).

### Poorly Differentiated Thyroid Carcinoma (PDTC) and Anaplastic Thyroid Carcinoma (ATC)

PDTC and ATC are aggressive tumors characterized by partial or total loss of differentiation, respectively. PDTCs show a poorer prognosis compared to WDTC, but a better 5-year overall survival (60-85%) compared to ATC (4, 21). For this reason, PDTCs are often considered morphologically and clinically halfway tumors between WDTC and ATC. In the past, a multistep evolution model for explaining thyroid cancer progression was proposed. According to this model, ATC and PDTC could derive directly from their well-differentiated counterparts, through the progressive acquisition of genetic alterations (5, 22, 23). More recently, the evidence derived from extensive molecular characterization of coexisting DTC and ATC, sustains the hypothesis of an early, independent evolution of tumor clones (24). It can be hypothesized that some ATCs emerge after a molecular and morphological evolution of DTCs, while others have a DTC-uncorrelated origin.

PDTC and ATC commonly harbor *BRAF* and *RAS* mutations, and often these mutations coexist with secondary genetic alterations, such as *PIK3CA*, *TP53* and *TERT* promoter mutations (9). However, the typical molecular frame of PDTC and ATC is different. For instance, PDTCs are more frequently gene fusion-driven tumors, while in ATCs gene fusions are rare. In addition, ATCs show a significantly higher prevalence of mutations in *TP53* gene (22, 23, 25, 26).

There are no dedicated treatment protocols for PDTC; thyroidectomy followed by RAI therapy offers a good local disease control, but the main cause of death is the metastatic spread (21, 27). Since PDTC show a relatively high frequency of gene rearrangements, molecular testing for potentially targetable alterations could offer important therapeutic options.

For patients with ATC, treatment options can have both therapeutic and palliative purposes. Tumor resection is considered depending on the extent of local invasion. In patients with systemic disease, thyroidectomy can help avoid future complication (i.e. airway obstruction). In unresectable tumors, neoadjuvant radiotherapy and/or chemotherapy can be considered (28). The use of chemotherapy regimens as systemic therapy for disease control highly depends on the established goals of care. Since targeted treatments are available for ATC patients, the recent American Thyroid Association guidelines recommend that molecular screening is performed at the time of diagnosis, so that clinicians can rapidly plan the most appropriate therapeutic strategy (28).

### Medullary Thyroid Carcinoma (MTC)

MTC is a rare neuroendocrine tumor originating from parafollicular cells. MTCs can be either hereditary or sporadic (20-25% and 75-80% of all cases, respectively) (29); histologically, there is no morphological difference between the two forms, but familial MTCs show more frequently c-cell hyperplasia and tumor multifocality (30). Hereditary MTC can be diagnosed either alone (familial MTC) or as part of the spectrum of Multiple Endocrine Neoplasia (MEN) type 2 syndromes, which are characterized by the presence of germline mutations in *RET* gene. Therefore, all inherited MTCs are virtually *RET*-driven (31). In sporadic MTCs, *RET* mutations are detected at somatic level in about 50-60% of cases, while *RAS* mutations have a prevalence of about 20-30% (31, 32).

MTC treatment and prognosis highly depend on disease stage at presentation. When there is no evidence of systemic involvement, thyroidectomy represents an effective strategy to minimize the risk of local recurrence (31). The extent of surgery (central and lateral compartment dissection) is established upon ultrasound findings and serum calcitonin levels. In locally advanced and metastatic setting, thyroidectomy is considered after careful cost-benefit evaluation; systemic therapies can help the control of disease. In children at risk of developing MTC (i.e. *RET* carriers), a prophylactic thyroidectomy can be also considered (31).

The algorithm for molecular diagnostics can be different for hereditary and sporadic MTC. It has to be specified that patients with suspected hereditary or familial MTC should be subjected to germline *RET* testing and genetic counselling. Also patients with presumed sporadic MTC should be referred to germline *RET* testing since, although rarely, they could be actually affected by hereditary forms (31). In case of advanced unresectable or metastatic MTC with germline *RET* testing not available or negative, a molecular screening should be performed on tumor tissue to search for somatic *RET* mutations (33).

## Targeted Therapy for Thyroid Cancer

In oncology, the terms “targeted therapy” indicate the use of anti-cancer drugs that target specific proteins involved in cancer growth and survival. The goal of these treatments is to inhibit tumor cell proliferation, with limited effect on normal cells.

Inhibitors that act on multiple kinases (multi-TKIs), i.e., nonselective targeted drugs, have been FDA-approved for treatment of both medullary and non-medullary thyroid cancer patients in advanced/metastatic setting. Efficacy of these inhibitors has been demonstrated in several clinical trial in terms of improved progression-free survival compared to placebo (5, 34).

Although predictive biomarkers of response to these drugs have been proposed and are still under investigation, no molecular characterization of tumors is needed before treatment. In fact, no clear association has been demonstrated between the presence of *BRAF*/*RAS* mutations and drug efficacy (4).

FDA-approved nonselective inhibitors for treatment of thyroid cancer are shown in **Table 1** (https://www.fda.gov, last accessed on 07/03/2022). Most of these agents not only act as inhibitors on tumor growth and proliferation, but they have also antiangiogenic effects.

**Table 1:** List of nonselective TKIs that are currently approved by FDA for the treatment of advanced thyroid cancer patients.

Drug name	Agent type	Main targets	Biological effects	Indication
Vandetanib	Multi-TKI	*EGFR*, *VEGFR*	Inhibition of tumor growth and angiogenesis	Unresectable locally advanced/metastatic MTC
Cabozantinib	Multi-TKI	*MET*, *VEGFR*, *AXL*, *RET*, *ROS1*, *TYRO3*, *MER*, *KIT*, *TRKB*, *FLT-3*, *TIE-2*	Inhibition of metastasis, angiogenesis, and maintenance of tumor microenvironment	Progressive, metastatic MTC; locally advanced/metastatic RAI-refractory DTC progressing following VEGFR-targeted therapy
Sorafenib	Multi-TKI	BRAF*, *KIT*, *FLT3*, *RET*, *VEGFR*, *PDGFRB*	Inhibition of tumor cell signaling, angiogenesis and apoptosis	Locally recurrent/metastatic, progressive RAI-refractory DTC
Lenvatinib	Multi-TKI	*VEGFR*, *FGFR*, *PDGFRA*, *KIT*, *RET*	Inhibition of angiogenesis, tumor growth and progression	Locally recurrent/metastatic, progressive, RAI-refractory DTC

TKI, tyrosine kinase inhibitor; MTC, medullary thyroid cancer; RAI, radioactive iodine; DTC, differentiated thyroid cancer.

*Including mutant BRAF

On the other hand, in the context of appropriate clinical indications, there are targeted drugs that act selectively on specific altered molecules, and thus are administered only if the tumor harbors specific molecular alterations. In other words, the presence of certain molecular characteristics makes the patient eligible for a specific treatment. In this case, molecular testing of tumor become mandatory.

### Predictive Biomarkers in Thyroid Cancer: Must-Test Genes

In this paragraph, targeted drugs currently approved for patients harboring specific genetic alterations are treated. According to OncoKB database (https://www.oncokb.org, last accessed on 17/03/2022), the number of actionable alterations in thyroid cancer is very limited. In detail, genes associated with predictive alterations with level of evidence 1, i.e., FDA approved drug in the specific indication, are listed in **Table 2** (https://www.fda.gov). There are no actionable genes with level of evidence 2 (biomarkers that are not FDA-recognized but indicated as predictive of response to an FDA-approved therapy by clinical guidelines).

**Table 2:** List of selective targeted drugs FDA-approved for thyroid cancer treatment.

Targeted agent	Target	Predictive marker	Thyroid cancer histotype*
Dabrafenib and Trametinib in combination	*BRAF*, *MEK*	*BRAF* p.V600E	locally advanced/metastatic ATC
Selpercatinib, Pralsetinib	*RET*	*RET* mutation	Advanced/metastatic MTC
		*RET* fusion	Advanced/metastatic, RAI-refractory thyroid cancer
Entrectinib**, Larotrectinib	*TRKA*, TRKB, *TRKC*	*NTRK* fusion	Unresectable/metastatic tumor progressing following prior treatment (tissue-agnostic)
Pembrolizumab	*PD-1*	MSI-H, TMB-H	Unresectable/metastatic tumor progressing following prior treatment (tissue-agnostic)

*As indicated by the FDA; includes tumor-agnostic drugs.

**Entrectinib has also activity on ROS1 and ALK receptors.

The predictive alteration and the specific indication for drug administration are also indicated. ATC, anaplastic thyroid cancer; MTC, medullary thyroid cancer; RAI, radioactive iodine; DTC, differentiated thyroid cancer; MSI-H, microsatellite instability – high; TMB-H, tumor mutational burden – high.

*BRAF* inhibitors such as dabrafenib and vemurafenib have been largely investigated in the treatment of advanced thyroid cancer. The efficacy of these agents in DTC patients seems promising, especially in the light of the overall high frequency of *BRAF* mutations in follicular-derived thyroid cancer. Nonetheless, the only histotype with a specific anti-*BRAF* ^V600E^ approved inhibitor is ATC in locally advanced or metastatic setting. In *BRAF* ^V600E^-driven ATC patients, dabrafenib in combination with trametinib, a MEK inhibitor, has shown 69% overall response rate (35).

Selective *RET* inhibitors have been approved for advanced *RET*-altered thyroid cancer and non-small cell lung cancer. These inhibitors seem highly effective in the treatment of medullary and non-medullary thyroid cancers, and they also show acceptable safety profiles (36–40).

The approval of drugs for tumor-agnostic treatment, virtually accessible to all patients with advanced stage tumors, highlighted that molecular characterization of tumor is becoming increasingly important for patients’ management. The agnostic approval of *NTRK*-rearranged selective inhibitors has derived from two clinical trials demonstrating their safety and efficacy in terms of response rate in several solid tumors (41–43).

When considering predictive marker testing, it is necessary to specify that molecular screening should be guided also by (i) the prevalence of a predictive alteration in each tumor type and (ii) the evidence of drug efficacy in a specific clinical setting. For example, treatment of thyroid cancer with NTRK selective inhibitors seems to induce potent and durable responses (44, 45). *NTRK1* and *NTRK3* fusions are detected in 2-4% of thyroid tumors in adults, while *NTRK2* fusion have never been described (9).

As regards immunotherapy targeted agents, data on treatment efficacy in thyroid cancer patients is limited. Response rates in patients with advanced DTC and ATC for the anti-PD-1 monoclonal antibody drug (pembrolizumab) monotherapy are generally low (46–48). Currently, several phase II clinical trials evaluating immunotherapy agents in thyroid cancer, including pembrolizumab monotherapy, are ongoing (49–51).

As regards immunotherapy predictive biomarkers, it is known that thyroid tumors generally do not show a high tumor mutational burden (TMB-H) and high microsatellite instability (MSI-H) (23, 52–54). For ATC, it has been hypothesized that response to pembrolizumab is independent of MSI status and TMB (55). Alternative predictive biomarkers to immunotherapy response have been largely investigated. Specifically, the expression of PD-L1 assessed by immunohistochemistry represents a useful biomarker for other cancer subtypes, but, also in this regard, evidence on its predictive role in DTC and ATC are limited (56).

Another crucial aspect to consider for immunotherapy is that, currently, the EMA does not recommend using pembrolizumab in a tumor-agnostic setting (https://www.ema.europa.eu, last accessed on 07/03/2022). For all these reasons, testing of TMB and MSI status is not always performed in thyroid cancer.

### Selective Inhibitors in Thyroid Cancer: New Clinical and Molecular Challenges

Although targeted drugs demonstrated highly effective in the treatment of advanced thyroid cancer, there are several issues that deserve further discussion.

First, targeted agents such as TKIs have generally positive effects on patients’ survival, but they do not allow a full recovery. Duration of response is always limited, and drug resistance cannot be avoided; the development of acquired resistance to TKI treatment invariably causes disease progression. The available data are related to resistance mechanisms emerging after TKI therapy in other tumor types, mainly in non-small cell lung cancer. It is known that molecular mechanisms causing resistance to selective TKIs can either activate alternative signaling pathways – off-target alterations – or directly interfere with drug binding – on-target alterations. In *RET*-rearranged non-small cell lung cancer, both types of resistance mechanisms have been described in patients treated with *RET*-inhibitors (i.e., *RET* mutations, *KRAS* and *MET* amplification) (56, 57). In *RET*-mutant MTC patients, acquired *RET* mutations have been associated with resistance to selpercatinib (57–59). Similarly, resistance mutations in *NTRK* genes emerging following targeted therapy have been described in several cancer types (60, 61). In thyroid cancer patients treated with selective BRAF inhibitors, the emergence of acquired mutations in RAS genes has been described as a resistance mechanism (62).

The development of resistance mechanisms represents an important challenge not only for clinicians, but also in molecular pathology. The laboratory should provide clinicians with rapid and usable results: to limit tissue re-biopsy, the use of liquid biopsy should be implemented; multi-target, highly sensitive methodologies should be used; laboratories should be able to test also uncommon alterations, such as copy number variations and tumor microenvironment alterations. In fact, the identification of resistance mechanisms can guide strategies to counteract cancer progression (63). In lung cancer, the emergence of resistance mutations in plasma can predict disease progression even before it becomes clinically evident (64).

The second most important downside of TKIs is the not negligible portion of patients experiencing adverse events. Although TKIs show lower toxicity profiles than conventional systemic therapies, adverse effects affecting multiple organs have been reported (5, 65, 66). The main adverse events include hypertension, gastrointestinal toxicity, fatigue; the most common endocrine-related toxicity affects thyroid function and thyroid hormone metabolism (67). The clinical spectrum of toxicities is variable, from minimal side effects manifestations to severe toxicities that lead to treatment discontinuation. Highly selective TKIs seems to show lower toxicity profiles compared to nonselective TKIs, likely due to a reduced off-target activity (38, 68).

### Targetable Alterations Outside of Approved Indication

Molecular testing can reveal the presence of predictive alterations for targeted drugs that are approved in other settings. If a targeted drug is approved for tumor type “A”, treatment is virtually inaccessible to patients with tumor type “B”, even though the specific predictive alteration is detected. In case of advanced, progressing thyroid tumors with no satisfactory alternative therapies, patients with predictive alterations can be enrolled either in clinical trials or in compassionate programs – if available, and if the inclusion criteria are met. Evidence of response rates of thyroid cancer patients following targeted drugs administration outside of approved indications mostly derives from case reports. In this context, treatment with *ALK* inhibitors in *ALK*-rearranged thyroid cancer patients showed promising results in terms of response rate and disease control (19, 69, 70).

A prospective, non-randomized clinical trial (NCT02925234) is enrolling patients with potentially targetable alterations who have exhausted alternative treatment options. This trial can be defined as a pan-cancer multi-drug program, in which patients carrying a druggable alteration have access to the specific targeted agent (71). The aim of the protocol is to evaluate response to treatment and survival rates of several anti-cancer drugs in different settings. The results of this clinical trial are likely to influence future tumor-agnostic drugs approval.

## Testing Methodologies and Platforms

Predictive biomarkers can be tested by a variety of methodologies and platforms. Commercial products both designed for thyroid cancer and pan-cancer panels are available. To give a short but practical overview on this topic, testing platforms can be divided according to the number and type of targets that should be tested. First, the number of actionable alterations in thyroid cancer is very limited, as already mentioned. Therefore, samples do not need particularly extensive molecular characterization. Moreover, the molecular landscape of thyroid tumors consists in mutually exclusive driver alterations that recur in few genes (12). In this regard, a cost-effective strategy can be represented by a first level analysis including *BRAF* and *RAS* genes, followed by further analyses in case of negative results. The cost-effectiveness of this kind of strategy compared to a deeper, multi-target molecular characterization performed up front highly varies according to different institutions. In fact, it is influenced by the number of samples, available facilities, type of gene panels, reimbursement policies and many other factors. Moreover, it has to be highlighted that an extensive molecular characterization could provide information on uncommon but targetable alterations (72). In advanced MTCs with negative or unavailable germline *RET* testing, the search for *RET* mutations should be conducted on tumor tissue; *RAS* mutations can be also detected in these tumors (33). Since it is advisable to analyze the entire coding region of *RET* gene, a next-generation sequencing (NGS) analysis can be more suitable compared to single target techniques.

Considering the type of predictive alterations, the recently approved targeted drugs against rearranged *RET* and *NTRK* genes complicated the picture of must-test alterations. Gene rearrangement analysis can be performed at three different levels: a) on DNA, by using NGS platforms, or *in-situ* methodologies; b) on RNA, with reverse-transcription PCR, by using specific primer pairs, NGS, or digital counting systems (nCounter nanoString); c) on protein, with immunohistochemistry (IHC) analysis, by using specific antibodies detecting an aberrant protein expression. Moreover, techniques can be either one-gene-one-test (such as IHC and fluorescent *in situ* hybridization, FISH) or multi-gene (NGS, nCounter system). Each technique has advantages and limitations. A screening based on IHC analysis would be extremely useful to select samples deserving molecular confirmation. However, in thyroid cancer, IHC could be considered not sufficiently accurate for *RET* and *NTRK* rearrangements (33, 73, 74). FISH represents a valuable *in situ* technique. The use of break-apart probes allows to identify a fusion event with no prior knowledge of the involved partner. It requires one tissue section per gene; therefore, it could be a sub-optimal choice in case of multiple targets to investigate. Moreover, FISH could miss small/intrachromosomal rearrangements, which are relatively frequent in *RET* gene (75). Several commercial RT-PCR tests have been validated for diagnostic use. Usually, specific primer-probe pairs are designed to anneal directly on the breakpoint region. The possibility to include probe pairs detecting multiple rearrangements in a single assay is technically limited, therefore several assays are necessary to cover the main fusion events. Therefore, FISH and RT-PCR could miss important information, but represent valuable instruments when NGS analysis is not available or not successful.

In molecular pathology, NGS analysis is becoming increasingly widespread. NGS is a high throughput technique that can be performed both on DNA and RNA. Several gene panels, also validated for diagnostic use, and different testing platforms are available. Accordingly, the number of targets is variable, but virtually NGS allows to analyze simultaneously different types of clinically relevant alterations, including single nucleotide variations, deletions, insertions and also gene fusions. Therefore, NGS represents a valuable resource for the analysis of all the required predictive biomarkers in thyroid cancer. Nonetheless, the analysis of rearrangements by NGS presents some disadvantages. For instance, DNA-based panels could miss rearranged cases compared to RNA-based ones (76). This is mainly due to the involvement of large intronic regions that make rearrangement detection technically challenging. Moreover, DNA analysis does not give information on the functionality nor on the expression of the fusion. NGS analysis performed on RNA can overcome these issues, but in turn presents an important technical limitation: it requires good quality RNA. RNA purified from formalin-fixed paraffin-embedded tissue is generally highly fragmented. Poor RNA quality can cause sequencing failure and also false negative results. In this regard, optimization of pre-analytical processing of tissue specimens is highly required (77, 78).

## Conclusions

During the last 10 years, the therapeutic strategies for patients with advanced thyroid cancer have been expanded thanks to the availability of new, effective targeted drugs. In this setting, the molecular screening of tumors has acquired a crucial role in the management of patients, as also highlighted by the introduction of agnostic drugs, namely agents that are effective on tumors carrying a specific genetic alteration, regardless of cancer histotype.

**Figure 2:**
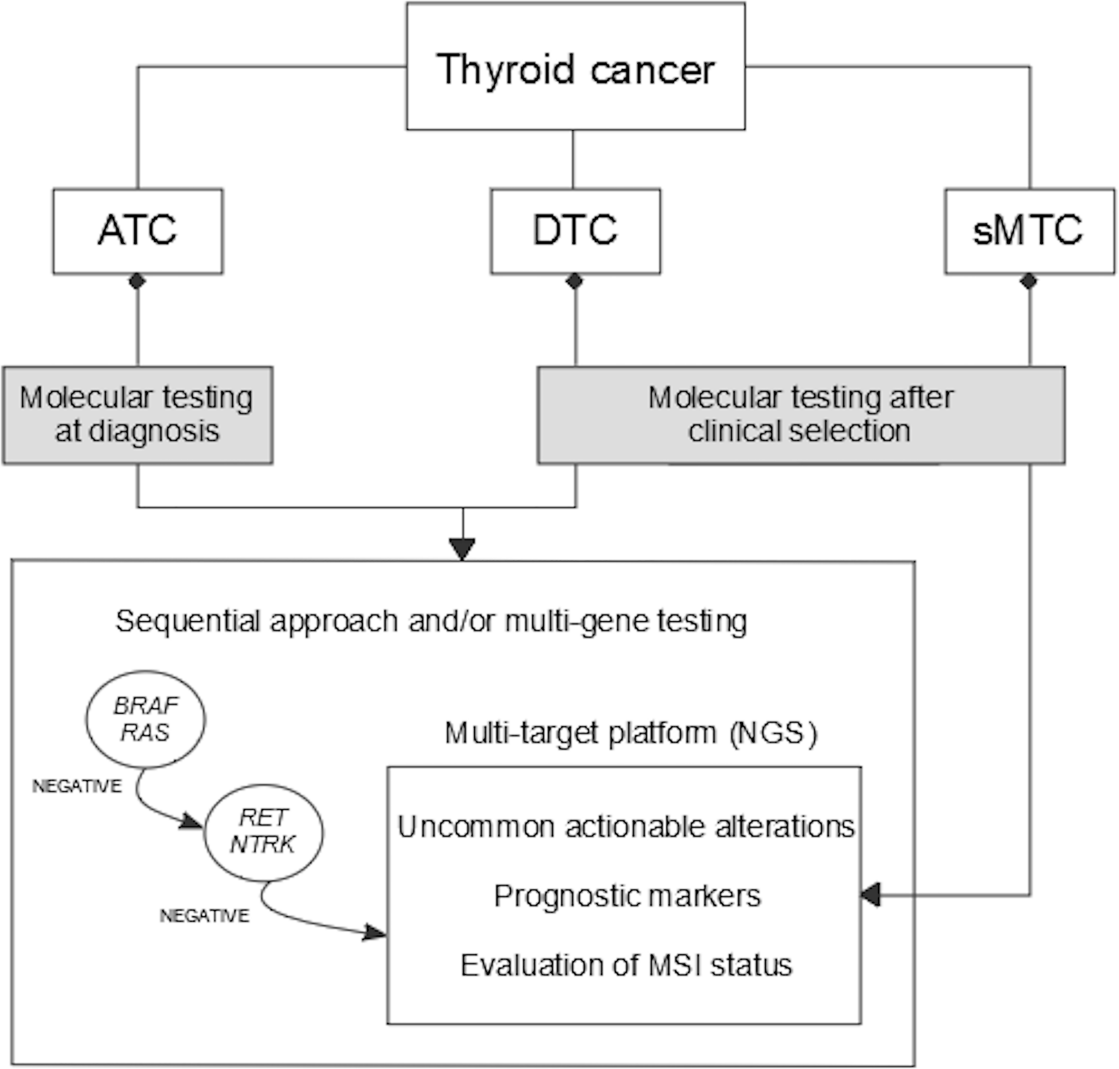
Algorithm for molecular diagnostics of predictive markers in thyroid cancer. ATC, anaplastic thyroid cancer; DTC, differentiated thyroid cancer; sMTC, sporadic medullary thyroid cancer; MSI, microsatellite instability; NGS, next-generation sequencing.

Herein, a global view of predictive biomarkers analysis in thyroid cancer is given. An overall summary of which, where and when predictive biomarkers should be tested is illustrated in **Figure 2**. A molecular screening including the must-test alterations should be always performed in patients who are clinically eligible for targeted drug treatment. In addition, an extensive molecular analysis of tumors can be performed to eventually reveal the presence of uncommon actionable alterations, which might offer relevant therapeutic options.

However, as highlighted in the present review, for a not negligible portion of patients who lack the main targetable molecular alterations, treatment alternatives have been not significantly improved. The investigation of novel targeted agents and a better identification of escape pathways involved in drug resistance can help overcome the current limitations of targeted treatments.

## Author Contributions

Conceptualization, EM and FB; literature search, EM, AMP, PV, and AB; data analysis, EM, AMP, PV, AP, CU, LT, and AB; data curation, all authors; writing, original draft preparation, EM; manuscript review and editing, EM, AMP, PV, and AB; supervision, FB, FS, and RE. All authors have read and approved the content of the manuscript.

## Conflict of Interest

The authors declare that the research was conducted in the absence of any commercial or financial relationships that could be construed as a potential conflict of interest.

## Publisher’s Note

All claims expressed in this article are solely those of the authors and do not necessarily represent those of their affiliated organizations, or those of the publisher, the editors and the reviewers. Any product that may be evaluated in this article, or claim that may be made by its manufacturer, is not guaranteed or endorsed by the publisher.
